# Population based registry study on large B-cell lymphoma mortality and morbidity in Finland

**DOI:** 10.2340/1651-226X.2025.42539

**Published:** 2025-02-25

**Authors:** Anna Anttalainen, Liisa Ukkola-Vuoti, Ville Vihervaara, Saija Silvola, Outi Kuittinen

**Affiliations:** a Medaffcon Oy, Espoo, Finland; b Takeda Oy, Helsinki, Finland; c Department of Oncology, Kuopio University Hospital Cancer Center, Kuopio, Finland; d Faculty of Medicine, Institute of Clinical Medicine, University of Eastern Finland, Kuopio, Finland

**Keywords:** LBCL, DLBCL, comorbidities, survival, mortality

## Abstract

**Background:**

Large B-cell lymphomas (LBCLs) form a notable subgroup of lymphomas; however, their associated long-term comorbidities and mortality rates remain under-researched in real-world settings.

**Material and methods:**

This nationwide Finnish population-based matched cohort study included virtually all LBCL patients (*N* = 7,019) diagnosed from 2008 to 2019, alongside age, sex, and region-matched controls (1:1 ratio) without lymphoma. Diagnoses of LBCLs were obtained from the Finnish Cancer Registry, with data linked to additional nationwide registries. Baseline characteristics were summarised using descriptive statistics. Overall survival (OS) was estimated using the Kaplan-Meier method, while Cox regression was used to analyse factors associated with OS and evaluate the risk and associated factors of comorbidities considering the competing risk of death.

**Results:**

The 5-year survival rate for LBCL patients, median age 70.7 years and 52.7% male, was 50.0% (95% Confidence Interval [CI] 48.7% – 51.3%), compared to 82.6% (95% CI 81.5% – 83.6%) for controls. Among LBCL patients, older age and a higher Charlson comorbidity index were associated with increased mortality. Conversely, female sex, later diagnosis year, and radiation therapy were associated with improved survival. Patients with LBCL exhibited an elevated risk of long-term comorbidities, including solid tumours, hematological and skin cancers, lung and thyroid diseases, mental and behavioral disorders, and cardiovascular diseases. After 12 years of follow-up, lymphoma accounted for the primary cause of death in approximately 43% of LBCL patients.

**Interpretation:**

Large B-cell lymphomas are linked with significant long-term comorbidities and elevated mortality rates.

## Population based registry study on large B-cell lymphoma mortality and morbidity in Finland

**Figure:**
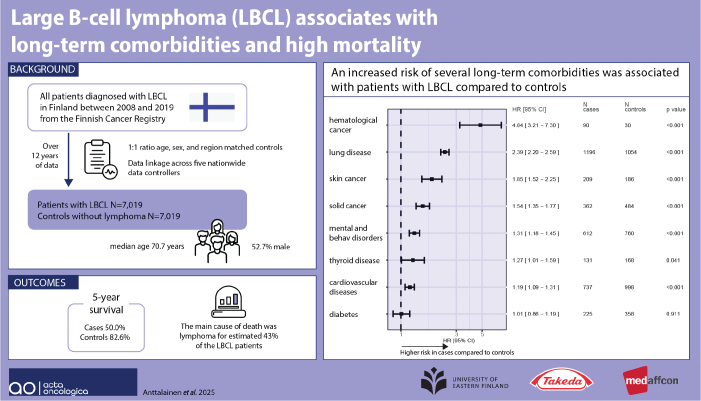

## Introduction

The current classification system for lymphomas encompasses several large B-cell lymphoma (LBCL) entities, among which diffuse large B-cell lymphoma not otherwise specified (DLBCL-NOS) is the most prevalent. This classification includes a heterogeneous group of clinically distinct diseases that exhibit varied responses to treatment and prognoses [1–3]. Although advancements in frontline treatments during the 2000s have improved the prognosis for LBCL, over one-third of patients continue to experience treatment-refractory or relapsed disease, ultimately leading to a less favourable prognosis [3–6]. Ten-year relative survival rates vary widely, ranging from 30% to 87%, largely reflecting differences in study populations and prognostic factors, including the stage of the disease at diagnosis and genetic findings [2, 4, 7, 8]. Achieving a 24-month event-free survival following diagnosis has been proposed as a critical endpoint for follow-up, as most lymphoma-related events occur before this milestone [6, 7, 9].

Despite improvements in overall survival (OS) over recent decades, LBCL continues to be associated with a substantial disease burden [10]. Additionally, the costs related to LBCL for both healthcare systems and patients remain high [11, 12]. However, long-term comorbidities associated with LBCL are understudied, and mortality among LBCL patients in Finnish real-world settings is not well understood. To address these gaps, we investigated OS and the primary causes of death in LBCL patients compared to control subjects, examining the associations of age, sex, diagnosis year, and Charlson comorbidity index (CCI) with OS. Our study aims to evaluate the long-term occurrence of complications and comorbidities associated with LBCL using a comprehensive nationwide population-based approach.

## Materials and methods

### Study design

This nationwide population-based cohort study included all Finnish individuals with newly diagnosed LBCL between January 01, 2008, and December 31, 2019. LBCL diagnoses were confirmed using the International Classification of Diseases for Oncology, Third Edition (ICD-O-3) morphology codes (detailed in Supplementary Methods). Data linkage across five nationwide data controllers – the National Institute of Health and Welfare (THL specialty care register, HILMO) [13], the Social Insurance Institution of Finland (SII), the Finnish Centre for Pensions, Statistics Finland, and the Digital and Population Data Services Agency (DPA) – was made possible via the Finnish personal identity number. The study adhered to the STROBE reporting guidelines.

LBCL patients were matched 1:1 with controls based on age, sex, and home municipality at the time of the LBCL diagnosis (hereafter referred to as the index date). Controls were selected from the DPA, ensuring they had no lymphoma diagnosis (C81–C85) from 1997 to 2019 and were alive at the case’s index date.

### Outcome ascertainment

LBCL -associated comorbidities were identified using THL registries through primary and secondary diagnoses recorded in specialty care, based on ICD-10 codes up to 3 years preceding the index date. Long-term comorbidities were categorised into eight groups: cardiovascular diseases (CVDs), mental and behavioral disorders, thyroid disease, diabetes, lung disease, hematological cancer, solid cancer excluding skin cancer, and skin cancer (Supplementary Methods). Radiation therapy (RT) was identified in the THL specialty care registry through procedure codes mentioning RT (‘sädehoito’) in the description (512 codes). Patients receiving RT within 1 year of the index date were classified as exposed to RT. Mortality data, including all-cause and cause-specific mortality categorised as deaths due to lymphoma, other cancers, CVDs, or other causes, were obtained from DPA and classified based on ICD-10 codes (Supplementary Methods) during 2008–2019. Central nervous system lymphomas were identified using ICD-O-3 topography codes C70.0–C72.9, and transformed lymphomas were defined using ICD-10 codes C82*, C83.00, or C83.80 as reported in HILMO prior to the LBCL index date.

### Statistical analyses

Summary statistics for patient demographics and clinical factors were presented as medians and interquartile ranges (IQRs) for continuous variables, and as counts and proportions for categorical variables. Differences between cases and controls were tested using McNemar’s test for categorical variables and the Wilcoxon signed-rank test for continuous variables, with *p*-values <0.05 considered statistically significant. Follow-up time was defined as the observed follow-up time from the index date to death or the study’s end, rather than using inverse Kaplan-Meier methods.

The CCI was calculated at baseline (up to 3 years before the index date) with a 6-month washout period, excluding lymphomas, using a modified scoring system [14]. Baseline CCI were categorised as 0, 1–2, and ≥3. Given the increased healthcare engagement for LBCL patients around diagnosis, they are more likely to have underlying comorbidities diagnosed. Thus, the washout period was implemented to ensure comparable baseline demographics between groups. The most prevalent baseline comorbidities (from 3 years to 6 months before the index date) were identified using ICD-10 diagnoses in specialty care. Differences were analysed with McNemar’s test.

OS was assessed using Kaplan-Meier analysis, with follow-up extending to the study’s end (31 December 2019) or death. Censoring was applied to patients alive at follow-up cessation. Differences in survival were tested using the log-rank test. Conditional survival analysis was conducted for LBCL patients who survived at least 24 months, limiting the subgroup further to those with their controls also alive at 24 months. The multivariable Cox regression model assessed the association of age, sex, diagnosis year, CCI, and radiotherapy status (considered as a time-dependent variable to avoid immortal time bias) with OS.

Cox regression models with two outcomes (comorbidity/death) were used to estimate the relative risk (hereafter referred to as risk) of long-term comorbidities associated with LBCL compared to controls. Comorbidity events were defined as the first diagnosis within pre-defined groups during the study period, including 3 years before the index date. Only hazard ratios (HRs) for comorbidity incidence are provided. A clustering variable for matched case-control pairs maintained the matched analysis. Diagnoses within 3 years before and 3 months after the index were defined as baseline, with the latter timeframe excluding comorbidities identified during increased healthcare contact. Individuals, with or without prevalent baseline comorbidities, were followed up until death or study completion. Aalen-Johansen estimators illustrated the estimated probabilities of patients being in each state (alive or dead, with or without comorbidity) over time, including those with baseline comorbidities.

Factors associated with comorbidities were analysed using Cox models with two outcomes (comorbidity/death), including only LBCL patients. Examined factors included sex, age at diagnosis, diagnosis year, CCI, and radiotherapy as a time-dependent variable. Aalen-Johanssen models were used to estimate competing risk probabilities for primary causes of death over time for both LBCL and control subjects. Patient counts below five were excluded from reporting to maintain data privacy. Missing values were not imputed. Statistical analyses were performed using R version 4.0.3.

## Results

**Figure 1:**
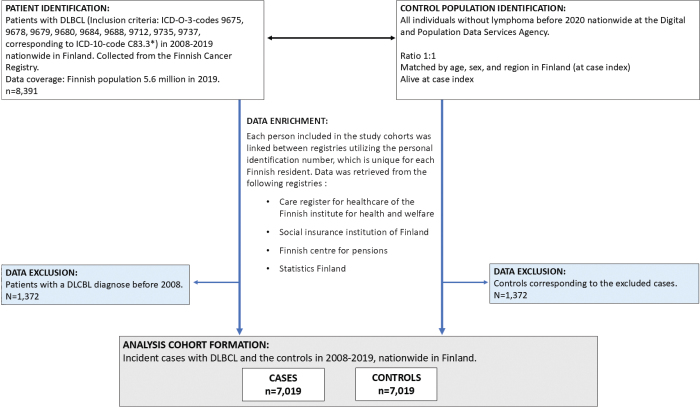
Cohort formation flowchart. Detailing excluded data segments (highlighted in blue boxes). ICD-O-3: International Classification of Diseases for Oncology, Third Edition; LBCL: Large B-Cell Lymphoma. *Denotes any symbol.

The study included a total of 7,019 LBCL patients (subgroups detailed in Supplementary Table 1) and their matched controls (*n* = 7,019; 52.7% male; median age 70.7 years) (Figure 1 and Table 1). Age and sex distributions were equivalent between patients and controls. LBCL patients had a significantly shorter median follow-up time than controls (1.7 vs. 4.4 years, *p* < 0.001). At baseline, LBCL patients exhibited higher CCI (CCI 0: 78.3% in cases vs. 83.4% in controls, CCI 1–2: 18.1% vs. 13.7%, CCI ≥3: 3.6% vs. 2.9%, *p* < 0.001). A total of 22.1% (*n* = 1,548) of LBCL patients received RT within 1 year of diagnosis, and 13.1% (*n* = 918) received it within 6 months. The most prevalent baseline co-occurring diagnoses included essential hypertension (patients 11.8%; controls 10.4%, *p* = 0.0084), atrial fibrillation and flutter (patients 7.6%; controls 6.2%, *p* = 0.0014), and senile nuclear cataract (patients 5.1%; controls 4.2%, *p* = 0.0072) (Supplementary Table 2).

**Table 1:** Baseline characteristics.

Variable	Cases (*n* = 7,019)	Controls (*n* = 7,019)	*P*-value
Median age at diagnosis [IQR]	70.72 [61.67, 79.08]	70.72 [61.67, 79.08]	1.000
Median follow-up years [IQR]	1.70 [0.37, 5.18]	4.41 [2.05, 7.38]	< 0.001
Male, *n* (%)	3,698 (52.7)	3,698 (52.7)	1.00
Charlson comorbidity index, pre-index, *n* (%)			
0	5,494 (78.3)	5,851 (83.4)	< 0.001
1–2	1273 (18.1)	961 (13.7)	
3 or over	252 (3.6)	207 (2.9)	
Radiotherapy within year from diagnosis *n* (%)	1,548 (22.1)	-	-
Radiotherapy within 6 months from diagnosis *n* (%)	918 (13.1)	-	-

IQR: Interquartile range.

### OS and risk factors

**Figure 2:**
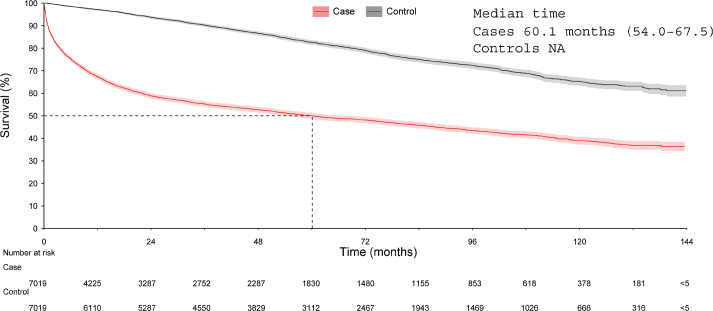
Overall survival in cases with large B-Cell Lymphoma and controls.

The OS for LBCL patients was significantly lower compared to controls (*p* < 0.001; Figure 2). The median OS for LBCL patients was 5.0 years [95% CI [confidence interval] 4.5–5.6], while the control group did not reach median OS within the study period. One-year survival rates were 67.4% [95% CI 66.2–68.5] for patients and 97.1% [95% CI 96.7–97.5] for controls. Five-year survival rates for patients and controls were 50.0% [95% CI 48.7–51.3] and 82.6% [95% CI 81.5–83.6], respectively; 10-year survival rates were 39.0% [95% CI 37.3–38.8] and 65.3% [95% CI 63.5–67.1], respectively (Supplementary Table 3).

For LBCL patients surviving past 24 months, the survival rate was 58.5% (93.3% among controls) (Supplementary Table 3). The conditional OS for these patients (*n* = 3,287; median age at diagnosis 66.3 years; 53.6% male, 26.0% received RT during the first year) is illustrated in Supplementary Figure 1A. While the conditional median OS was not reached, the 5-year survival rate for this subgroup was 77.9% [95% CI 76.1% – 79.5%], and the 10-year survival rate was 61.9% [95% CI 58.4% – 65.1%] (Supplementary Table 4). Further analysis for patients with both case and control surviving the 24-month landmark (*n* = 3,147; median age at diagnosis 65.9 years; 53.4% male; 26.2% received RT during the first year) is presented in Supplementary Figure 1B. The 5-year survival rate was 78.9% [95% CI 77.1–80.6] for patients and 86.6% [95% CI 85.3–88.2] for controls, with 10-year survival rates of 63.3% [95% CI 59.8–66.6] and 74.8% [95% CI 71.7–77.6], respectively (Supplementary Table 4), with differences in OS being statistically significant (*p* < 0.0001).

**Figure 3:**
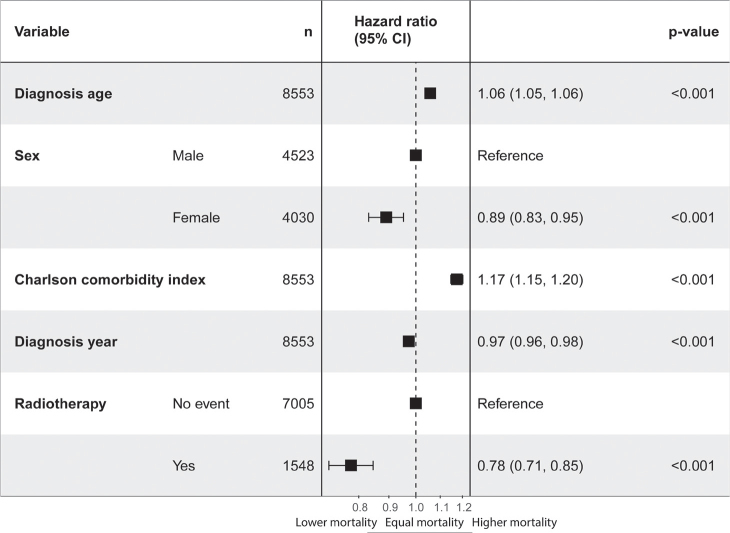
Cox proportional hazards models for the association of diagnosis age, sex, Charlson comorbidity index, diagnosis year, and radiotherapy on Large B-Cell Lymphoma patient survival. The hazard ratios (HR) with 95% confidence intervals (CI) and p-values are reported.

Higher mortality was associated with older age at diagnosis (HR 1.06 per year [95% CI 1.05–1.06], *p* < 0.001) and higher CCI (HR 1.20 [95% CI 1.17–1.24], *p* < 0.001). Factors associated with improved survival included female sex (HR 0.89 [95% CI 0.83–0.95], *p* < 0.001), later diagnosis year (HR 0.97 per year [95% CI 0.96–0.98], *p* < 0.001), and RT within the first year post-diagnosis (HR 0.78 [95% CI 0.72–0.85], *p* < 0.001) (Figure 3). However, the proportional hazards assumption was not met for RT status, and the HR represents an average risk over time.

### Risk of long-term comorbidities

**Figure 4:**
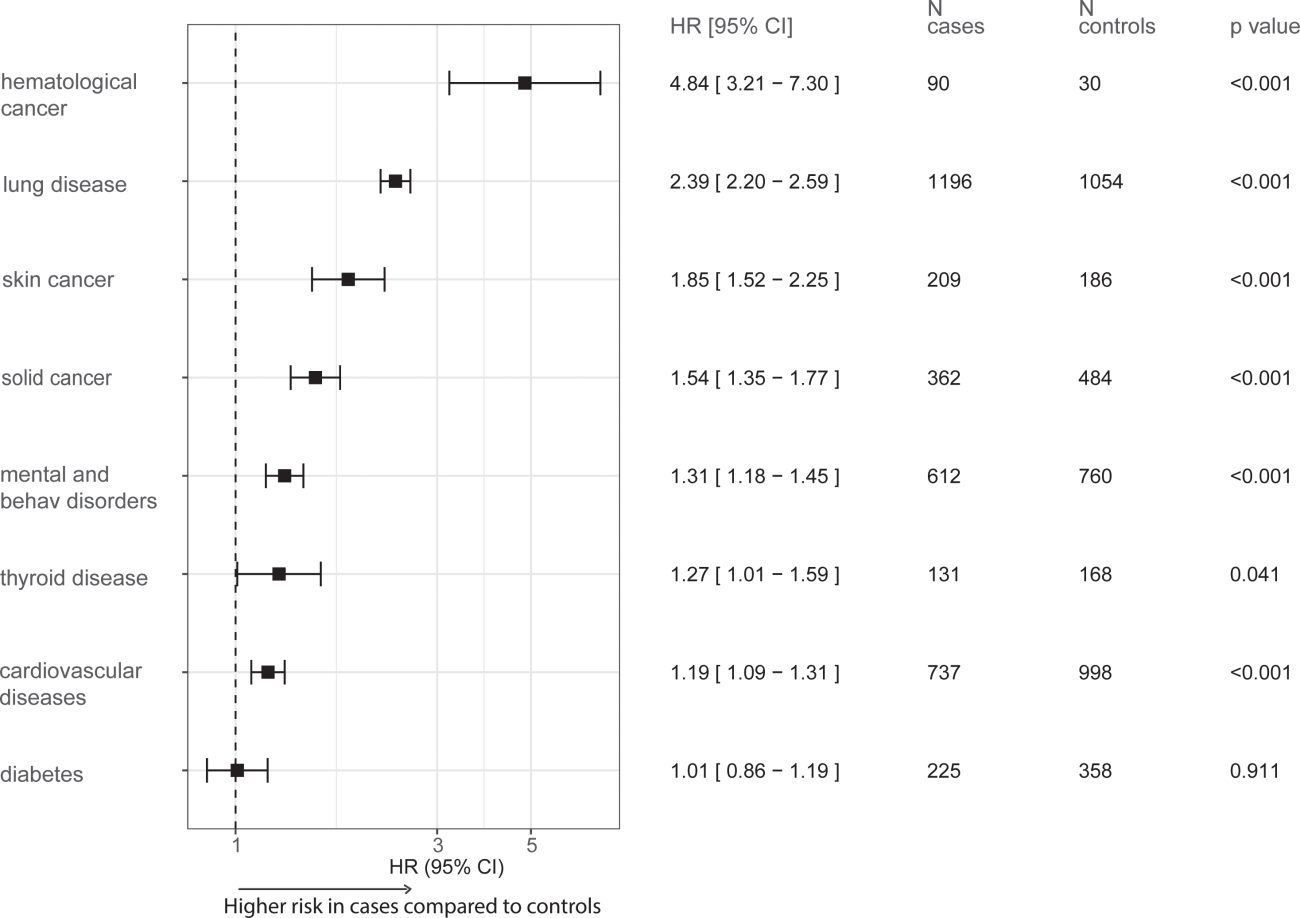
Hazard ratios (HR) with 95% confidence intervals (CI) for the relative risk of long-term comorbidities during the follow-up period in Large-B-Cell Lymphoma patients compared to controls. Number of new comorbidity diagnoses during the follow-up period is presented. Data for competing risk of death is not shown.

Comparative analysis demonstrated that LBCL patients experienced a higher risk of most preselected long-term comorbidities than controls (Figure 4). Significant increases were noted for hematological cancer (HR = 4.84 [95% CI 3.21–7.30], *p* < 0.001), lung disease (HR = 2.39 [95% CI 2.20–2.59], *p* < 0.001), skin cancer (HR = 1.85 [95% CI 1.52–2.25], *p* < 0.001), solid cancer (HR = 1.54 [95% CI 1.35–1.77], *p* < 0.001), mental and behavioral disorders (HR = 1.31 [95% CI 1.18–1.45], *p* < 0.001), thyroid disease (HR = 1.27 [95% CI 1.01–1.59], *p* = 0.041), and CVDs (HR = 1.19 [95% CI 1.09–1.31], *p* < 0.001). No significant association was found between LBCL and increased diabetes risk. The proportional hazards assumptions were violated for all comorbidities except hematological cancer, skin cancer, and thyroid disease due to non-linear incidence over time. Despite this, the presented HRs approximate the average risk accurately. Patient state proportions over time are depicted in Supplementary Figure 2.

Older age at diagnosis was associated with increased risk of lung disease (HR = 1.01 per year [95% CI 1.01–1.02], *p* < 0.005), solid cancer (HR = 1.03 [95% CI 1.02–1.04], *p* < 0.005), mental and behavioral disorders (HR = 1.02 [95% CI 1.02–1.03], *p* < 0.005), CVDs (HR = 1.06 [95% CI 1.05–1.06], *p* < 0.005), diabetes (HR = 1.03 [95% CI 1.02–1.04], *p* < 0.005), and skin cancer (HR = 1.07 [95% CI 1.06–1.08], *p* < 0.005) (Supplementary Table 5). Higher CCI was linked with increased risks of lung disease (HR = 1.10 [95% CI 1.02–1.18], *p* = 0.0173), mental and behavioral disorders (HR = 1.20 [95% CI 1.10–1.30], *p* < 0.005), and CVDs (HR = 1.18 [95% CI 1.08–1.28], *p* < 0.005) (Supplementary Table 5). Female patients had a higher risk of mental and behavioral disorders (HR = 1.32 [95% CI 1.13–1.55], *p* < 0.005) and thyroid disease (HR = 2.92 [95% CI 2.00–4.26], *p* < 0.005), whereas they exhibited decreased risks for lung disease (HR = 0.84 [95% CI 0.75–0.94], *p* < 0.005), solid cancer (HR = 0.67 [95% CI 0.54–0.83], *p* < 0.005), CVDs (HR = 0.78 [95% CI 0.67–0.90], *p* < 0.005), diabetes (HR = 0.56 [95% CI 0.42–0.73], *p* < 0.005), and skin cancer (HR = 0.56 [95% CI 0.42–0.73], *p* < 0.005) compared to males (Supplementary Table 5). The risk for solid (HR = 0.94 per year [95% CI 0.90–0.97], *p* < 0.005) and hematological cancer (HR = 0.92 [95% CI 0.85–0.99], *p* = 0.0301) diminished over successive years of diagnosis (Supplementary Table 5). RT exposure was not significantly associated with the risk for any studied comorbidities (Supplementary Table 5). Competing risk factors for death are not displayed. Proportional hazard assumptions were met for all variables except for RT concerning the risk of death.

### Cumulative causes of death

**Figure 5:**
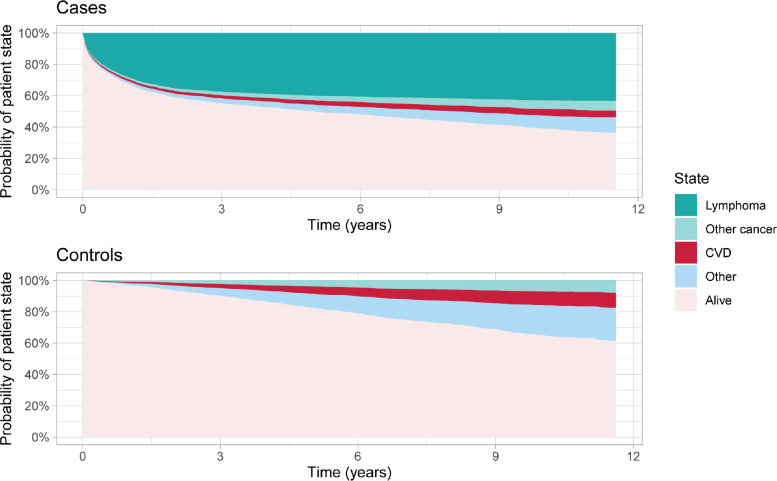
Causes of and the proportions of deaths among cases with Large B-Cell Lymphoma and corresponding controls. CVD: cardiovascular diseases

The cumulative proportion of LBCL patients dying from lymphoma was 28.4% at 1 year and increased to 35.3% at 2 years post-diagnosis. Subsequently, lymphoma-related deaths decreased, reaching a cumulative proportion of 43.2% at 12 years (Figure 5, Supplementary Table 6). Initially, the cumulative proportion of deaths from CVDs was higher among patients (1.3%) than controls (0.9%) at 1 year. However, the cumulative proportion of CVD-related deaths was greater among controls (4.7%) than patients (2.7%) at 5 years, continuing to rise over time. The cumulative proportion of deaths from other cancers was generally higher among controls (7.7%) than patients (6.1%) at 12 years post-diagnosis (Figure 5, Supplementary Table 6).

## Discussion

To our knowledge, this study is the first to analyse LBCL-associated mortality and long-term comorbidities using Finnish nationwide real-world data over a 12-year follow-up. During this period, over 40% of cases succumbed to lymphoma, reflecting a higher mortality rate for LBCL patients than previously reported. Our study revealed a median OS of 5.0 years for LBCL patients, contrasting with the Surveillance, Epidemiology and End Results (SEER) database from the United States, which reported a median survival of 6.3 years for the 1997–2015 cohort [15]. Additionally, a median OS of 6.0 years was reported within the Hospital District of Southwest Finland [16]. The disparity may stem from our inclusion of post-mortem diagnoses (*survival of these patients was from 0 to 1 days, n=77; 0.11% of the patients*), as well as elderly patients with comorbidities who did not receive active anti-lymphoma therapy. Our cohort also encompassed primary central nervous system lymphomas (PCNSL; 6.6%) and transformed lymphomas (4.2%), both of which generally exhibit poorer outcomes compared to *de novo* non-CNS DLBCL [17, 18].

The classification of lymphomas is complex, encompassing over 100 entities, and has progressively evolved to be more biologically representative. Currently, the 2022 World Health Organization (WHO) fifth edition of haematolymphoid tumour classification (WHO-HAEM5) includes 17 LBCL entities, with DLBCL not otherwise specified (NOS) being the most common [19]. Historical registry analysis poses challenges due to varying lymphoma classifications over the study period (12 years). Initially, the 1997 WHO II classification included only two of the current LBCL entities [20]. Furthermore, the adoption of new classifications by hospitals and pathologists varied. Discrepancies in ICD-O-3 and ICD-10 coding, relative to WHO classifications, further complicate the analysis. As a result, maintaining distinct subgroups would require extensive pathology review, an impractical task for such large datasets. Therefore, despite understanding data heterogeneity, we reported all LBCL types collectively, acknowledging that while this may affect treatment outcome evaluations, it should not significantly influence the risk of long-term comorbidities.

Achieving event-free survival at 24 months post-diagnosis (EFS24) is considered a surrogate marker of cure in DLBCL [6, 9]. However, a Swedish study across five counties found 20% of DLBCL patients surviving event-free for at least 24 months still died from lymphoma [7]. Unfortunately, our study focused on survival status due to a lack of remission data. In our study, 66% of patients who survived 24 months remained alive after 10 years, which is slightly lower than the previously reported 75% [7]. Furthermore, restricting the sub-population to those with controls also surviving at 24 months revealed significantly poorer survival among LBCL patients compared to controls, suggesting inclusion of non-cured and late-relapse cases among 2-year survivors.

Considering factors like age, CCI, female sex, and later diagnosis years, RT within 12 months of diagnosis was associated with improved survival in LBCL patients in our study. Nearly a quarter of our patients received RT, 59% of whom within 6 months of diagnosis. Although randomised trials evaluating post-immunochemotherapy RT are lacking, two Swedish registry studies imply survival benefits in selected patient populations [21, 22]. Conversely, Tokola et al. found no survival improvement, potentially due to selection bias as RT is administered to patients with more manageable disease or stable progression during induction [23]. While RT plays an essential role in treating lymphoma, its long-term adverse effects are well known [10]. In our study, increased comorbidity risks were not associated with RT, possibly due to the follow-up duration of up to 12 years (median 1.7 years) being insufficient to capture long-term complications. Additionally, the variability in RT sites in LBCL treatments could obscure a precise link between RT and long-term complications on a population level.

Our study also observed higher survival rates among females, consistent with findings in multiple lymphoma subtypes and other cancers [24, 25]. The underlying mechanisms remain unidentified, though sex hormones during fertile years are hypothesised to contribute. Notably, the improved relative survival of DLBCL patients diagnosed more recently, reported in the Netherlands, aligns with robust treatment advancements [26].

Our findings corroborate previous studies indicating lymphoma survivors face risks of secondary malignancies [8, 10, 27–29]. Despite their rarity, the most substantial comorbidity risk increase occurred with hematological cancers among both patients and controls. Moreover, LBCL patients exhibited elevated risks for skin and solid cancers, and more frequently diagnosed lung and thyroid diseases, potentially due to pulmonary toxicity from immunochemotherapy, growth factors [30], and radiation to neck and thoracic regions [10]. Enhanced CVD and mental health disorder risks were noted in LBCL patients, with prior studies indicating cognitive impairments impacting up to a third of DLBCL patients 8 years post-diagnosis [31–33]. This cognitive burden, alongside emotional, economic, and social stressors, likely contributes to heightened mental health risks. Diagnostic bias due to intensified healthcare interactions and monitoring among LBCL patients may lead to earlier comorbidity detection as opposed to controls, evident in our findings of higher skin cancer risks. Moreover, therapy-induced immunosuppression could accelerate cutaneous lesion progression. Nevertheless, these factors alone likely do not fully account for the higher observed comorbidity burden among patients. Although diabetes has been linked with Non-Hodgkin lymphoma (NHL) patients in the year following steroid-immunochemotherapy regimens [34], our study did not reveal higher diabetes risks post-LBCL diagnosis. Additionally, recent meta-analyses associated cholesterol-rich diets and obesity, known DLBCL risk factors, with improved patient survival; however, we lacked specific data on diet and obesity in our study [21].

### Strengths and limitations

This study boasts several strengths, notably its population-based design, minimising selection bias and enhancing the generalisability of the results. The unique Finnish personal identity number facilitated robust data linkage across high-quality health registries, offering comprehensive nationwide coverage and complete follow-up [35]. Finland’s tax-funded healthcare system ensures equitable access to public healthcare services for all residents, regardless of socioeconomic status, covering a population of 5.6 million as of 2019 [36]. The successful matching procedure ensured equivalent age and sex distribution between patients and controls. However, for 12 LBCL patients, regional matching was unattainable, necessitating a broader geographic match across Finland. Consequently, using a 1:1 case-to-control ratio was warranted.

Nevertheless, the study has limitations, primarily the absence of clinical data detailing disease presentation, treatment specifics, in-hospital drug administration, and chemotherapy dosages, alongside a lack of pathology review, compelling the use of a broad LBCL category. Due to insufficient treatment data, event-free survival could not be confirmed for conditional survival analyses of LBCL patients surviving beyond 24 months. Although prescription medication data reflect nearly complete nationwide coverage, our data was limited to reimbursed purchases. Despite 86.9% of LBCL patients not receiving RT within 6 months of diagnosis, this finding should be interpreted cautiously, as several factors could account for the absence of frontline RT, with primary refractory disease likely contributing to only a minority of cases. Information regarding RT site, dose and volume was unavailable.

## Conclusions

LBCL is associated with multiple long-term comorbidities and elevated mortality. Survival was adversely affected by older age and a higher number of comorbidities at diagnosis. In contrast, females exhibited a seemingly lower mortality risk compared to males, as did patients diagnosed more recently and those receiving RT. Nonetheless, no significant association between RT and long-term comorbidities was observed, potentially due to the follow-up period being too brief to capture these effects. Lymphoma-specific mortality was notable throughout the study, peaking within the first 2 years following LBCL diagnosis.

## Supplementary Material

Population based registry study on large B-cell lymphoma mortality and morbidity in Finland

## Data Availability

The dataset from this study is maintained within Statistics Finland’s secure operating environment, Fiona. Due to data sharing agreements, the dataset cannot be made publicly available. However, data and the underlying analysis plan can be requested from the corresponding author (Outi Kuittinen) and will be shared pending necessary permissions from Finnish authorities.
