# Supplementary material for: Population based registry study on large B-cell lymphoma mortality and morbidity in Finland
Source: Acta Oncol. 2025 Feb 25;64:42539. doi: 10.2340/1651-226X.2025.42539 (PMC11877859; doi:10.2340/1651-226X.2025.42539)
Supplement: Population based registry study on large B-cell lymphoma mortality and morbidity in Finland [file AO-64-42539-s1.pdf]

Supplementary material has been published as submitted. It has not been copyedited, or typeset by Acta Oncologica

Supplementary Materials

Population based registry study on large B-cell lymphoma mortality and morbidity in Finland

Anna Anttalainen *et al.* 2025.

Supplementary Methods

The ICD-O-3 morphology codes for LBCL were: 9675, 9678, 9679, 9680, 9684, 9688, 9712, 9735, 9737, corresponding to ICD10 diagnosis code C83.3.

The ICD10-codes for long term comorbidities were:

cardiovascular diseases (CVDs; ICD-10 codes: I21, I25, I50, I63, G45)

mental and behavioral disorders (F0-F3, F40-F48, F5, R53)

thyroid disease (E00-E07)

diabetes (E10-E14)

lung disease (J00-J06, J09, J10-J18, J20-J22, J3, J40-J47, J6, J70, J80-J86, J9)

hematological cancer (C90-C96, D46)

solid cancer excluding skin cancer (C0, C1, C20-C26, C3, C40, C41, C45-C49, C50-C58, C6, C7, C80, C97)

skin cancer (C43, C44)

The ICD10-codes for causes of death were:

lymphoma (C81-85)

other cancer (ICD-10: C0, C1, C20-C26, C3, C4, C50-C58, C6, C7, C80, C90-C97, D46)

CVDs (I21, I25, I50, I63, G45)

Supplementary Table 1: The number of large B-cell lymphoma (LBCL) patients with morphology code (ICD-O-3) subgroup.

ICD-O-3	Definition	n
9680	Malignant lymphoma, large B-cell, diffuse, NOS	6816
9679	Mediastinal (thymic) large B-cell lymphoma	86
9688	T-cell rich large B-cell lymphoma	58
9684	Malignant lymphoma, large B-cell, diffuse, immunoblastic, NOS	29
9735	Plasmablastic lymphoma	15
9712	Intravascular large B-cell lymphoma	11
9678	Primary effusion lymphoma	<5
9675	Malignant lymphoma, mixed small and large cell, diffuse	
9737	ALK positive large B-cell lymphoma	

Supplementary Table 2: The most commonly reported baseline comorbidities of cases and controls recorded during 3 years prior to LBCL diagnosis.

ICD-10	Diagnosis	Cases, n (%)	Controls, n (%)	p
I10	Essential (primary) hypertension	825 (11.8)	729 (10.4)	**0.0084**
I48	Atrial fibrillation and flutter	531 (7.6)	436 (6.2)	**0.0014**
H25.1	Senile nuclear cataract	358 (5.1)	292 (4.2)	**0.0072**
I25.1	Atherosclerotic heart disease	287 (4.1)	271 (3.9)	0.504
E11.9	Non-insulin-dependent diabetes mellitus - Without complications	273 (3.9)	236 (3.4)	0.1056
H90.3	Sensorineural hearing loss, bilateral	254 (3.6)	253 (3.6)	1
N40	Hyperplasia of prostate	228 (3.2)	188 (2.7)	0.0437
J18.9	Pneumonia, unspecified	210 (3)	183 (2.6)	0.1794
I50.9	Heart failure, unspecified	176 (2.5)	154 (2.2)	0.2375
R10.4	Other and unspecified abdominal pain	182 (2.6)	158 (2.3)	0.2013
C61	Malignant neoplasm of prostate	155 (2.2)	165 (2.4)	0.5946
M17.1	Other primary gonarthrosis	164 (2.3)	153 (2.2)	0.5656
G47.3	Sleep apnoea	172 (2.5)	143 (2)	0.1054
R07.4	Chest pain, unspecified	151 (2.2)	161 (2.3)	0.6057
J45.9	Asthma, unspecified	142 (2)	124 (1.8)	0.2973
R42	Dizziness and giddiness	144 (2.1)	118 (1.7)	0.1167
Z01.0	Examination of eyes and vision	139 (2)	121 (1.7)	0.2765
Z01.8	Other specified special examinations	150 (2.1)	117 (1.7)	**0.0442**
Z03.9	Observation for suspected disease or condition, unspecified	146 (2.1)	112 (1.6)	**0.0209**
K57.3	Diverticular disease of large intestine without perforation or abscess	126 (1.8)	117 (1.7)	0.5986
L57.0	Actinic keratosis	151 (2.2)	89 (1.3)	**<0.001**
I25.9	Chronic ischaemic heart disease, unspecified	104 (1.5)	121 (1.7)	0.2818
I63.9	Cerebral infarction, unspecified	115 (1.6)	113 (1.6)	0.9465
M05.8	Other seropositive rheumatoid arthritis	162 (2.3)	62 (0.9)	**<0.001**
R06.0	Dyspnoea	117 (1.7)	108 (1.5)	0.5905
I70.2	Atherosclerosis of arteries of extremities	115 (1.6)	96 (1.4)	0.2065
M16.1	Other primary coxarthrosis	102 (1.5)	113 (1.6)	0.4912
M48.0	Spinal stenosis	100 (1.4)	108 (1.5)	0.6224
Z96.1	Presence of intraocular lens	117 (1.7)	98 (1.4)	0.1974
A46	Erysipelas	110 (1.6)	81 (1.2)	0.0395
Z01.1	Examination of ears and hearing	104 (1.5)	93 (1.3)	0.4646
H25.8	Other senile cataract	87 (1.2)	91 (1.3)	0.8148
M17.0	Primary gonarthrosis, bilateral	89 (1.3)	97 (1.4)	0.6038
N10	Acute tubulo-interstitial nephritis	111 (1.6)	75 (1.1)	**0.0087**
C44.31	Basal cell carcinoma of skin of other and unspecified parts of face	110 (1.6)	62 (0.9)	**<0.001**
J44.8	Other specified chronic obstructive pulmonary disease	94 (1.3)	71 (1)	0.0868
K40.9	Unilateral or unspecified inguinal hernia, without obstruction or gangrene	87 (1.2)	82 (1.2)	0.7555
M79.6	Pain in limb	90 (1.3)	81 (1.2)	0.5383
N39.0	Urinary tract infection, site not specified	81 (1.2)	93 (1.3)	0.3932
R55	Syncope and collapse	95 (1.4)	68 (1)	**0.0405**
A09	Diarrhoea and gastroenteritis of presumed infectious origin	83 (1.2)	66 (0.9)	0.1839
G30.1	Alzheimer's disease with late onset	55 (0.8)	94 (1.3)	**0.0013**
H26.4	After-cataract	89 (1.3)	72 (1)	0.2045
M54.5	Low back pain	75 (1.1)	83 (1.2)	0.5752
R53	Malaise and fatigue	77 (1.1)	84 (1.2)	0.6299
Z00.0	General medical examination	82 (1.2)	74 (1.1)	0.5597
Z71.8	Other specified counselling	76 (1.1)	73 (1)	0.8602

Supplementary Table 3: Kaplan-Meier estimates for overall survival (OS) among LBCL patients and their matched controls at different timepoints during the study period.

Time	N cases at risk	Estimate (%) for cases (95 % CI)	N controls at risk	Estimate (%) for controls (95 % CI)
1 month	6229	89.1 (88.4-89.8)	6957	99.8 (99.7-99.9)
2 months	5849	84.5 (83.7-85.4)	6889	99.6 (99.5-99.7)
3 months	5580	81.3 (80.4-82.2)	6812	99.4 (99.1-99.5)
6 months	5026	75.3 (74.3-76.3)	6577	98.6 (98.3-98.9)
1 year	4225	67.4 (66.2-68.5)	6110	97.1 (96.7-97.5)
2 years	3287	58.8 (57.6-60)	5287	93.7 (93.1-94.3)
3 years	2752	55.2 (54-56.4)	4550	90.3 (89.5-91)
4 years	2287	52.7 (51.4-54)	3829	86.5 (85.6-87.4)
5 years	1830	50 (48.7-51.3)	3112	82.6 (81.5-83.6)
6 years	1480	48.2 (46.8-49.5)	2467	79 (77.8-80.1)
7 years	1155	45.8 (44.4-47.2)	1943	75 (73.6-76.3)
8 years	853	43.4 (42-44.9)	1469	72.4 (70.9-73.8)
9 years	618	41.6 (40-43.1)	1026	68.8 (67.2-70.4)
10 years	378	39 (37.3-40.7)	666	65.3 (63.5-67.1)
11 years	181	36.8 (34.9-38.8)	316	63.2 (61.1-65.2)
12 years	<5	36.4 (34.3-38.5)	<5	61.1 (58.6-63.6)

Supplementary Table 4: Kaplan-Meier estimates for conditional overall survival (OS) among LBCL patients who survived past 24-month landmark (a patients surviving past 24-months; b cases and controls for LBCL patients alive and with control alive at 24 months).

	**a)**		**b)**			
	**LBCL patients**		**LBCL patients**		**controls**	
**Time from LBCL diagnosis**	**N at risk**	**Estimate (%) (95 % CI)**	**N at risk**	**Estimate (%) (95 % CI)**	**N at risk**	**Estimate (%) (95 % CI)**
25 month	3249	99.4 (99.1-99.6)	3110	99.4 (99.1-99.6)	3122	99.8 (99.6-99.9)
26 months	3202	98.8 (98.3-99.1)	3067	98.8 (98.4-99.2)	3089	99.6 (99.3-99.8)
27 months	3162	98.3 (97.8-98.7)	3030	98.4 (97.9-98.8)	3059	99.4 (99.1-99.6)
30 months	3024	96.9 (96.2-97.4)	2902	97.1 (96.4-97.6)	2957	99 (98.6-99.3)
3 years	2752	93.9 (93.0-94.7)	2651	94.3 (93.4-95.1)	2746	97.8 (97.2-98.2)
4 years	2287	89.6 (88.5-90.7)	2204	90.2 (89.1-91.3)	2332	95.4 (94.5-96.1)
5 years	1830	85 (83.6-86.3)	1762	85.7 (84.3-87)	1919	92.9 (91.8-93.8)
6 years	1480	81.9 (80.3-83.3)	1423	82.6 (81.1-84.1)	1550	89.8 (88.5-90.9)
7 years	1155	77.9 (76.1-79.5)	1119	78.9 (77.1-80.6)	1236	86.8 (85.3-88.2)
8 years	853	73.9 (71.9-75.8)	832	75.2 (73.2-77.1)	927	84.3 (82.6-85.9)
9 years	618	70.7 (68.5-72.7)	602	72 (69.8-74.1)	673	81.3 (79.3-83.1)
10 years	378	66.3 (63.7-68.7)	365	67.4 (64.8-69.9)	436	78.6 (76.3-80.7)
11 years	181	62.6 (59.6-65.6)	176	64.2 (61.1-67.1)	213	76.1 (73.4-78.6)
12 years	<5	61.9 (58.4-65.1)	<5	63.3 (59.8-66.6)	9	74.8 (71.7-77.6)

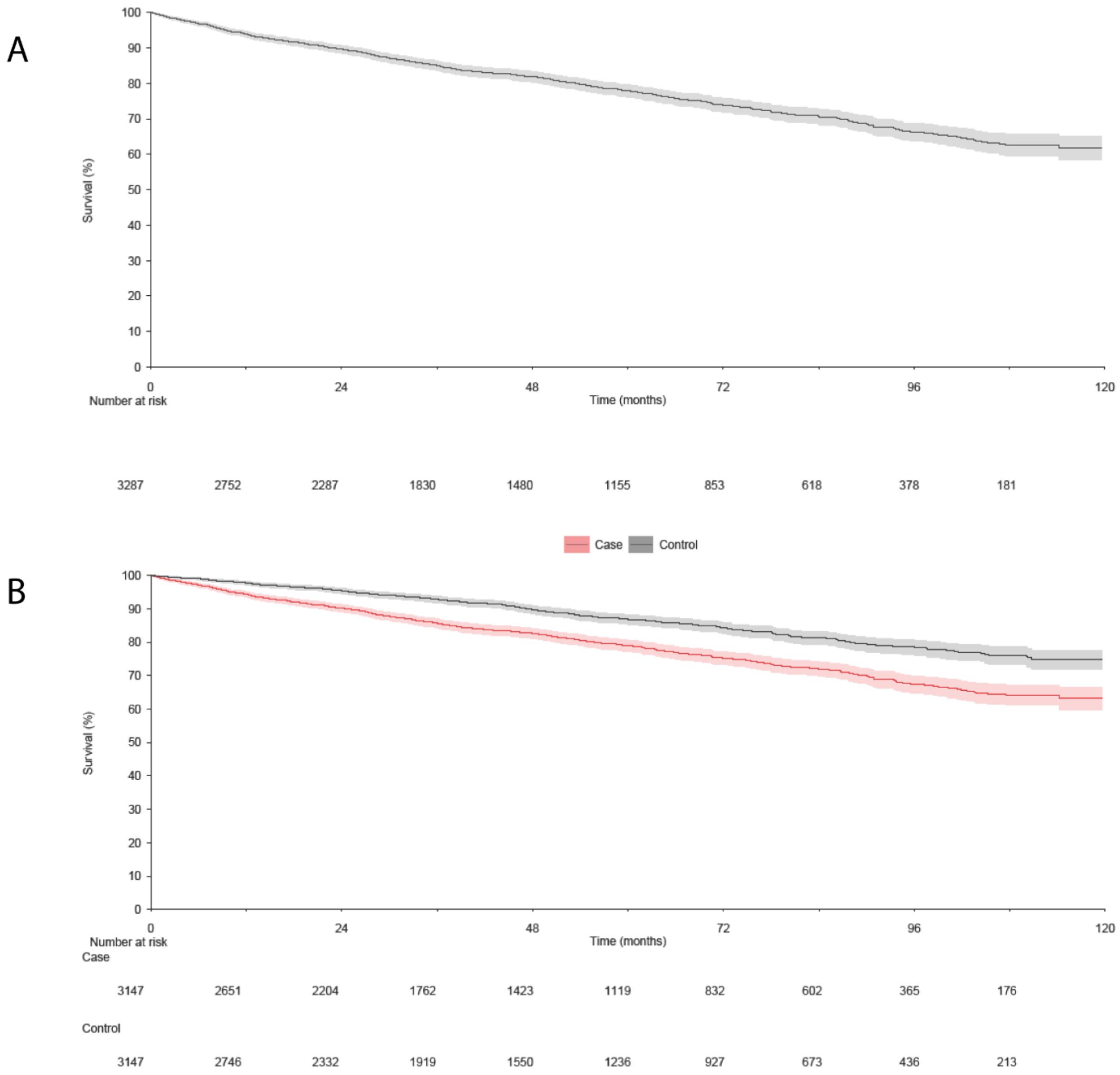

Supplementary Figure 1: Conditional overall survival (OS) a) among LBCL patients who survived past 24-month landmark and b) cases and controls, starting from 24 months after LBCL diagnosis.

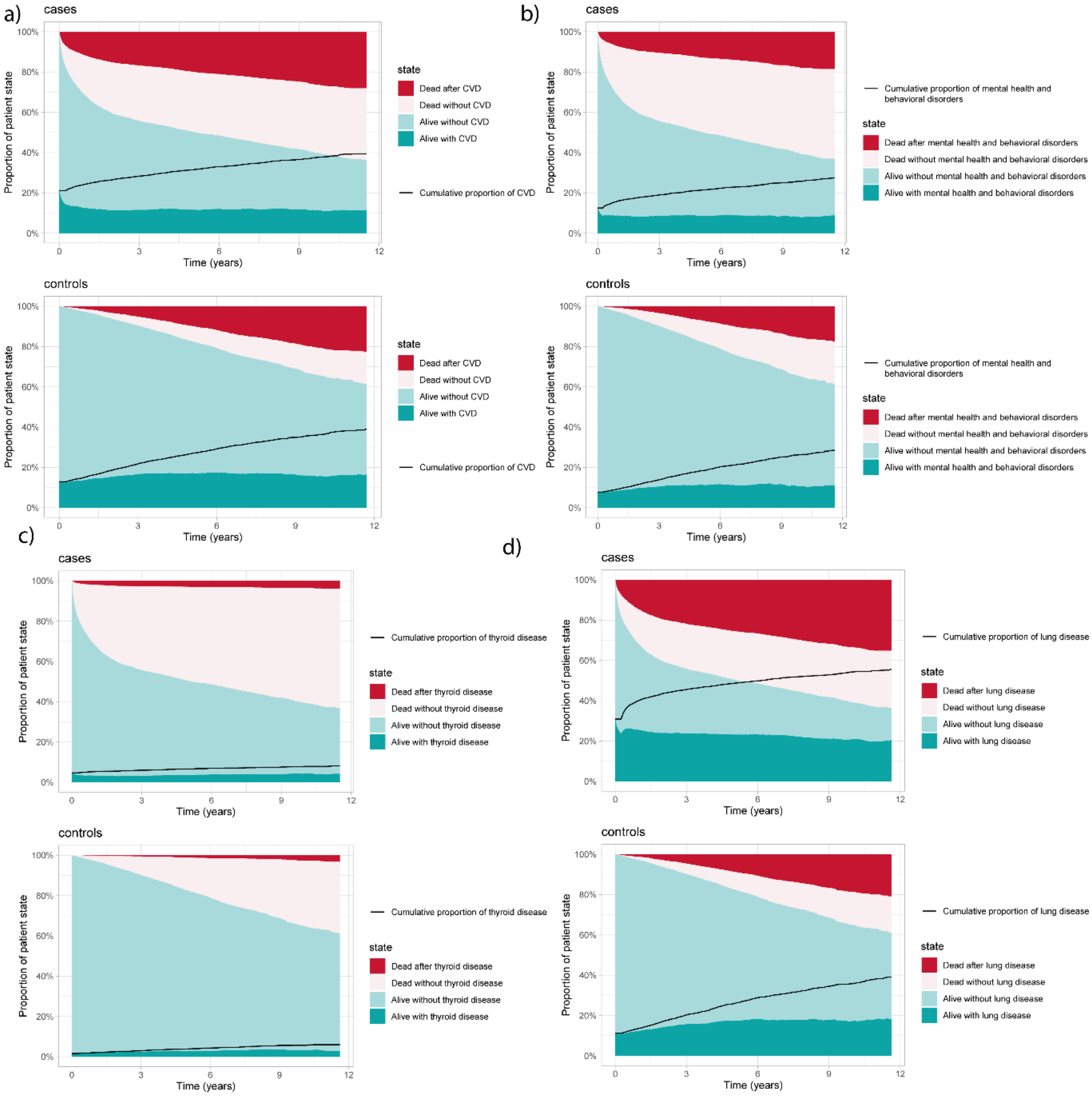
a)
cases
controls
Proportion of patient state
Time (years)
state
Dead after CVD
Dead without CVD
Alive without CVD
Alive with CVD
Cumulative proportion of CVD
b)
cases
controls
Proportion of patient state
Time (years)
Cumulative proportion of mental health and behavioral disorders
state
Dead after mental health and behavioral disorders
Dead without mental health and behavioral disorders
Alive without mental health and behavioral disorders
Alive with mental health and behavioral disorders
c)
cases
controls
Proportion of patient state
Time (years)
Cumulative proportion of thyroid disease
state
Dead after thyroid disease
Dead without thyroid disease
Alive without thyroid disease
Alive with thyroid disease
d)
cases
controls
Proportion of patient state
Time (years)
Cumulative proportion of lung disease
state
Dead after lung disease
Dead without lung disease
Alive without lung disease
Alive with lung disease

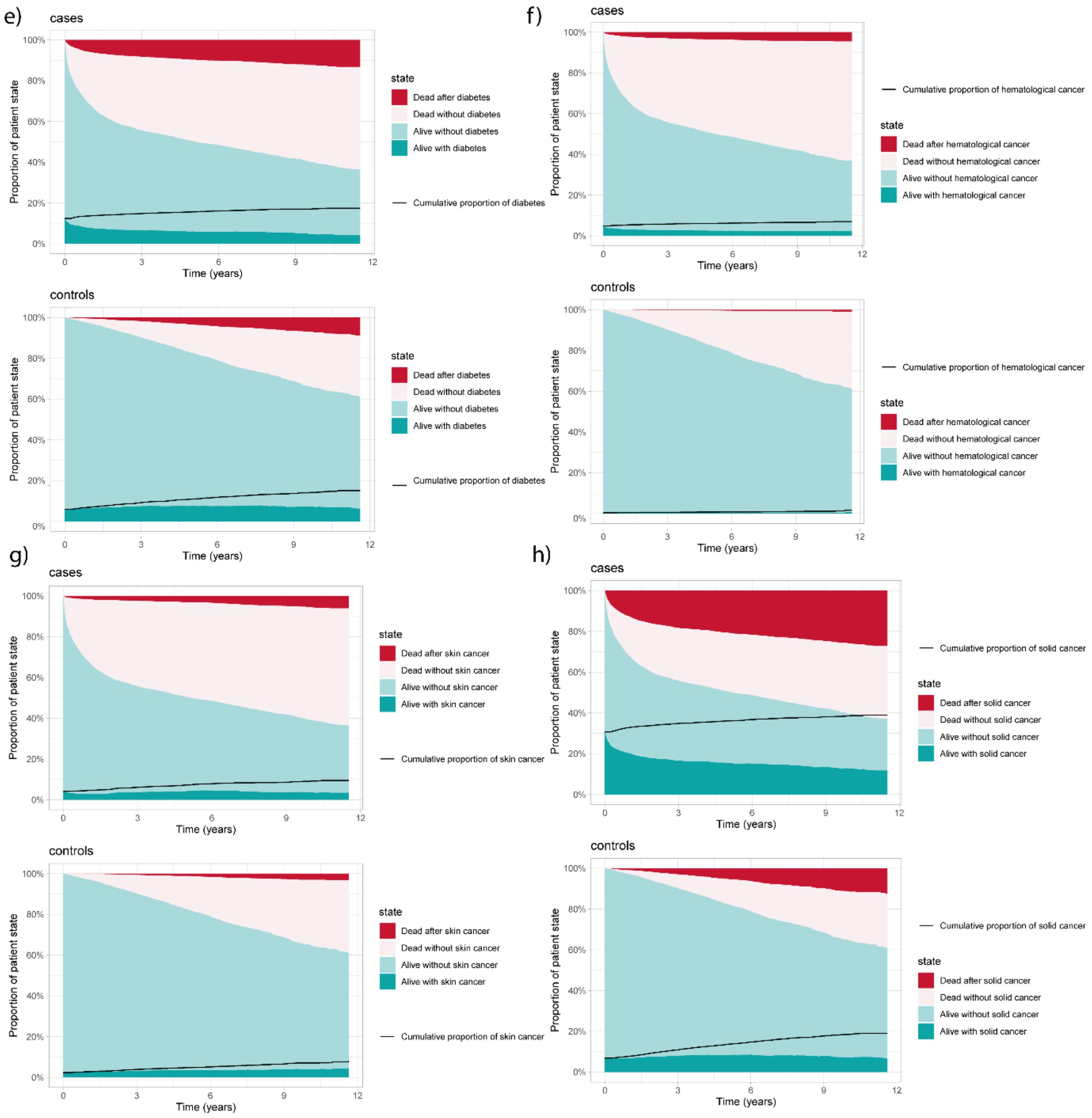

Supplementary Figure 2. Aalen-Johanssen estimates for proportions of patients in each state (a-h) during the study period, for LBCL patients and matched controls. Diagnoses during the 3 first months after LBCL diagnosis were included in the baseline diagnoses, observed as fixed cumulative proportion during this period.

Supplementary Table 5. Hazard ratios (HR) from univariable Cox proportional hazard models for covariates associated with the occurrence of long-term comorbidities.

	Age at diagnosis	CCI	Sex (female)	Diagnosis year	Radiated
**Lung disease**	**1.01 (1.01-1.02), p<0.005**	**1.07 (1.01-1.13), p=0.0273**	**0.84 (0.75-0.94), p<0.005**	0.99 (0.98-1.01), p=0.6037	0.89 (0.76-1.04), p=0.1309
**Solid cancer**	**1.03 (1.02-1.04), p<0.005**	1.09 (0.95-1.26), p=0.2289	**0.67 (0.54-0.83), p<0.005**	**0.94 (0.9-0.97), p<0.005**	1.02 (0.78-1.33), p=0.8961
**Mental health problems**	**1.02 (1.02-1.03), p<0.005**	**1.2 (1.12-1.28), p<0.005**	**1.33 (1.13-1.56), p<0.005**	1.01 (0.98-1.04), p=0.5569	0.92 (0.75-1.13), p=0.4188
**CVD**	**1.06 (1.05-1.06), p<0.005**	**1.12 (1.04-1.2), p<0.005**	**0.77 (0.67-0.9), p<0.005**	1.01 (0.98-1.04), p=0.527	1.01 (0.85-1.21), p=0.8825
**Hematological cancer**	1.01 (0.99-1.02), p=0.2774	0.87 (0.66-1.14), p=0.3075	1.06 (0.7-1.6), p=0.7975	**0.92 (0.85-0.99), p=0.0295**	0.81 (0.46-1.42), p=0.4534
**Thyroid disease**	1.01 (1-1.02), p=0.1375	1.14 (0.98-1.33), p=0.0931	**2.93 (2.01-4.28), p<0.005**	0.99 (0.93-1.06), p=0.8363	0.7 (0.44-1.12), p=0.1341
**Diabetes**	**1.03 (1.02-1.04), p<0.005**	1 (0.88-1.15), p=0.9634	**0.56 (0.42-0.73), p<0.005**	0.96 (0.92-1), p=0.0762	0.87 (0.61-1.23), p=0.4197
**Skin cancer**	**1.07 (1.06-1.08), p<0.005**	0.98 (0.85-1.13), p=0.761	**0.56 (0.42-0.74), p<0.005**	1.02 (0.96-1.07), p=0.5312	0.91 (0.65-1.27), p=0.5755

Supplementary Table 6. Cumulative causes and proportions of death and patients alive at specified time points among LBCL patients and controls during the study period.

Time (years)	Cause of death or state	Proportion estimate (cases)	Proportion estimate (controls)
1	Lymphoma	28.4 %	0 %
	Other cancer	1.3 %	0.8 %
	CVD	1.3 %	0.9 %
	Other	1.7 %	1.1 %
	Alive	67.4 %	97.1 %
2	Lymphoma	35.3 %	0 %
	Other cancer	1.8 %	1.5 %
	CVD	1.6 %	1.7 %
	Other	2.5 %	3.1 %
	Alive	58.8 %	93.7 %
3	Lymphoma	37.4 %	0 %
	Other cancer	2.3 %	2.3 %
	CVD	2 %	2.5 %
	Other	3.1 %	4.8 %
	Alive	55.2 %	90.3 %
4	Lymphoma	38.7 %	0 %
	Other cancer	2.7 %	3.2 %
	CVD	2.3 %	3.5 %
	Other	3.6 %	6.8 %
	Alive	52.7 %	86.5 %
5	Lymphoma	40 %	0 %
	Other cancer	3.1 %	3.8 %
	CVD	2.7 %	4.7 %
	Other	4.3 %	8.9 %
	Alive	50 %	82.6 %
10	Lymphoma	43 %	0.1 %
	Other cancer	5.5 %	7 %
	CVD	4.2 %	8.6 %
	Other	8.3 %	19 %
	Alive	39 %	65.3 %
12	Lymphoma	43.2 %	0.1 %
	Other cancer	6.1 %	7.7 %
	CVD	4.4 %	9.6 %
	Other	9.8 %	21.4 %
	Alive	36.4 %	61.1 %
